# The Subunit AEC/BC02 Vaccine Combined with Antibiotics Provides Protection in *Mycobacterium tuberculosis*-Infected Guinea Pigs

**DOI:** 10.3390/vaccines10122164

**Published:** 2022-12-16

**Authors:** Xiaonan Guo, Jinbiao Lu, Junli Li, Weixin Du, Xiaobing Shen, Cheng Su, Yongge Wu, Aihua Zhao, Miao Xu

**Affiliations:** 1 National Engineering Laboratory for AIDS Vaccine, School of Life Sciences, Jilin University, Changchun 130012, China; 2 Division of Tuberculosis Vaccine and Allergen Products, Institute of Biological Product Control, National Institutes for Food and Drug Control, Beijing 102629, China

**Keywords:** latent tuberculosis infection, chemotherapy, AEC/BC02 vaccine, immunotherapy, protection

## Abstract

A latent tuberculosis infection (LTBI) is a major source of active tuberculosis, and addressing an LTBI is crucial for the elimination of tuberculosis. The treatment of tuberculosis often requires a 6-month course of multidrug therapy, and for drug-resistant tuberculosis, a longer course of multidrug therapy is needed, which has many drawbacks. At present, vaccines are proposed as an adjunct to chemotherapy to protect populations with an LTBI and delay its recurrence. In this study, we analyzed the protective effect of a novel subunit vaccine, AEC/BC02, in a guinea pig latent infection model. Through the optimization of different chemotherapy durations and immunization times, it was found that 4 weeks of administration of isoniazid–rifampin tablets combined with three or six injections of the vaccine could significantly reduce the gross pathological score and bacterial load in organs and improve the pathological lesions. This treatment regimen had a better protective effect than the other administration methods. Furthermore, no drug resistance of *Mycobacterium tuberculosis* was detected after 2 or 4 weeks of administration of the isoniazid–rifampin tablets, indicating a low risk of developing drug-resistant bacteria during short-term chemotherapy. The above results provided the foundation for an AEC/BC02 clinical protocol.

## 1. Introduction

Tuberculosis is the 13th leading cause of death worldwide and the second leading cause of death from a single infectious agent after COVID-19 [1]. The World Health Organization (WHO) reported that there were about 10.6 million people living with tuberculosis worldwide in 2021, of which about 1.4 million HIV-negative and 187,000 HIV-positive individuals died of tuberculosis. As a result of the COVID-19 pandemic in 2019, years of global progress in reducing the number of deaths from tuberculosis have been reversed, bringing the total number of deaths in 2021 back to the levels reached in 2017 [1,2]. There are many challenges in the prevention and control of tuberculosis, such as a latent tuberculosis infection (LTBI), drug-resistant tuberculosis, and a co-infection with SARS-CoV-2 or HIV [3]. An LTBI is a subclinical mycobacterial infection with a cellular immune response to mycobacterial antigens but no clinical manifestations [4]. It is estimated that about a quarter of the global population is infected with *Mycobacterium tuberculosis* (*M.tb*), and studies have shown that more than 85% of active tuberculosis cases originated from an LTBI [5]. However, bacillus Calmette–Guerin (BCG), the only currently approved vaccine, can only provide effective protection in children, but not in adults, and it is not effective against an LTBI [6,7]. Therefore, the development of a new tuberculosis vaccine is urgently needed.

At present, chemotherapy is an important means to control the tuberculosis epidemic, as it eliminates the source of infection, thereby curing tuberculosis patients [8]. However, the drawbacks of a long-term anti-tuberculosis drug treatment includes potential adverse reactions and a poor compliance, which can result in the recurrence of tuberculosis and drug resistance [9,10]. Reducing the duration of chemotherapy to improve compliance, while ensuring the treatment’s effectiveness, is a challenge in the control of tuberculosis. In light of the challenges facing the use of anti-tuberculosis drugs, therapeutic vaccines have become the focus of more recent research [11]. The specific immune response induced by a vaccine is expected to promote the clearance of *M.tb*, consolidate the effects of chemotherapy, shorten the required duration of chemotherapy, improve the acceptance and completion rate of the treatment, reduce the possibility of drug resistance, and finally obtain a higher cure rate [12,13]. In 2019, the WHO summarized the development of tuberculosis therapeutic vaccines and issued the “Preferred product characteristics (PPC)” document, providing guidance for future research [14]. In preclinical studies, the PPC document suggested that animal model studies should be employed to support the application of therapeutic vaccines to improve the therapeutic effects of chemical drugs [15,16,17].

AEC/BC02, a new recombinant tuberculosis vaccine, is composed of the recombinant *M.tb* antigen Ag85b, ESAT6-CFP10 (EC), and a complex adjuvant system BC02, and has completed phase I clinical trials [18]. The AEC/BC02 vaccine demonstrated a good protective effect in a guinea pig latent infection model, reducing the degree of organ disease and the viable bacterial load in the spleen and lungs [19]. However, several questions regarding the dosage of a vaccine and the combinatorial effect of chemotherapy remained unanswered. In the current study, *M.tb*-infected guinea pigs were treated with short-term chemotherapy, followed by multiple injections of the AEC/BC02 vaccine for a therapeutic immunization. We evaluated the risk of a drug resistance during short-term chemotherapy and the immune protective effect of the AEC/BC02 vaccine in guinea pigs by a combination of different chemotherapy durations and vaccine doses. By exploring the potential of AEC/BC02 as an immunotherapeutic vaccine after infection, we hope to provide supporting data for clinical trials.

## 2. Materials and Methods

### 2.1. Animals and Bacteria

Female specific-pathogen-free Hartley guinea pigs, weighing 350–500 g, were purchased from the Institute for Laboratory Animal Resources of the China National Institute of Food and Drug Control (NIFDC). The animals were housed in an ABSL-3 laboratory at the Chinese Center for Animal Disease Control and Prevention. All the animals used in this study were treated according to animal welfare standards and the protocols were reviewed by the NIFDC Animal Care and Welfare Committee.

The *M.tb* strain CMCC (B) 95052, which is virulent in guinea pigs, was preserved at −70 °C and a bacterial suspension was prepared in our laboratory. As positive control bacteria, isoniazid and rifampicin double-resistant strains of CMCC 94024 were prepared in our laboratory.

### 2.2. Antibiotics and Vaccines

Antibiotic tablets (isoniazid–rifampin tablets), each containing 300 mg of rifampin (RFP) and 150 mg of isoniazid (INH), were manufactured by Shenyang Hongqi Pharmaceutical Company (Shenyang, China).

The recombinant tuberculosis vaccine AEC/BC02 was produced by the Anhui Zhifei Longcom Biologic Pharmacy Company (Hefei, China). One dose of AEC/BC02 vaccine was comprised of 10 μg of Ag85b and 10 μg of ESAT6-CFP10 in 0.5 mL of adjuvant BC02.

### 2.3. Study Design

**Figure 1:**
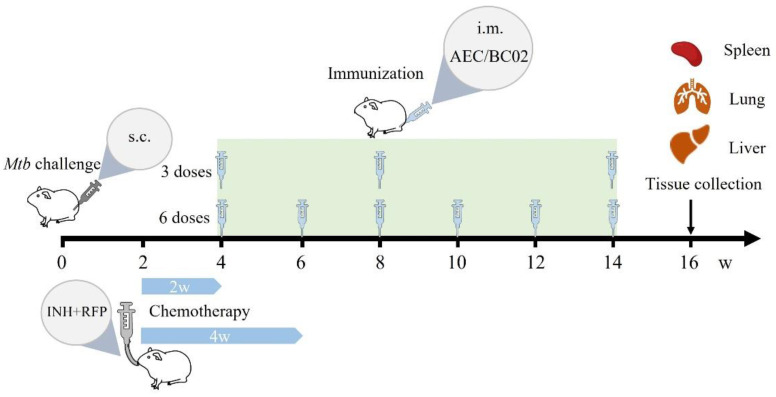
The optimal immunotherapy schedule for evaluation of a potential vaccine candidate in a *M.tb* latently infected guinea pig model. All guinea pigs were subcutaneously infected with *M.tb* into the groin for 2 weeks, followed by intragastric administration of INH + RFP for 2 or 4 weeks. Guinea pigs were injected intramuscularly with three or six doses of AEC/BC02 vaccine at 4 weeks after infection, and the animals were dissected at 2 weeks after immunization. The negative control group was injected with the same dose of saline during chemotherapy and immunization.

Fifty-four guinea pigs were divided into nine groups, as shown in Table 1. The experimental process is shown in Figure 1. In brief, the guinea pigs were infected with *M.tb* for 2 weeks and then administered anti-tuberculosis drugs by gavage for 2 or 4 weeks. After 4 weeks of *M.tb* infection, three or six doses of freeze-dried recombinant tuberculosis vaccine (AEC/BC02) were injected. Two weeks after the last immunization, the guinea pigs were dissected to observe the degree of pathological changes in the liver, spleen, and lungs of the guinea pigs, to isolate and culture the viable bacteria in the spleen and lungs, to detect the drug resistance of the culture, and to examine the histopathology of the liver, spleen, and lungs. The groups of guinea pigs comprised the immunotherapy group (group 1–group 4), the chemotherapy groups (group 5 and group 6), the vaccine groups (group 7 and group 8), and the negative control group (group 9).

### 2.4. Guinea Pig Infection

The *M.tb* bacterial suspension that had been stored at −70 °C was thawed at room temperature, then diluted to 1 × 10^4^ CFU/mL with normal saline. Each guinea pig was administered 0.5 mL of the diluted bacterial suspension subcutaneously into the groin, thereby receiving a theoretical infection dose of 5000 CFU. At the same time, the challenge bacteria were serially diluted and inoculated into a Lowenstein–Jensen (L–J) culture media at 37 °C for 4 weeks. The results of the viable counts showed that the actual infection dose was 8000 CFU per animal.

### 2.5. Chemotherapy

After grinding one isoniazid–rifampin tablet into a fine powder, 30 mL of 1% starch solution was added and mixed well. The RFP content was 10 mg/mL and the INH content was 5 mg/mL. According to Table 1 and Figure 1, the administration started 2 weeks after the *M.tb* infection in the guinea pigs, twice a week, and the corresponding groups were administered chemotherapy by gavage for 2 or 4 weeks.

### 2.6. Vaccination

According to Figure 1 and Table 1, after 4 weeks of *M.tb* infection (2 weeks of chemotherapy), the guinea pigs in the corresponding group were intramuscularly injected with 0.5 mL of one human dose of vaccine in the hind leg. A total of three or six injections were given, of which three injections were given at 4, 8, and 14 weeks after infection, and six injections at 4, 6, 8, 10, 12, and 14 weeks after infection.

### 2.7. Observation of General Condition

During the whole experimental period, the general condition of the animals was observed and the animals were weighed every week. Any weight changes in the animals were analyzed.

### 2.8. Gross Pathological Score and Bacterial Loads

All the animals were euthanized and dissected at 16 weeks after *M.tb* infection, and the liver, spleen, and lung were scored by a lesion index according to previous scoring criteria (Table 2) [20]. The sum of the scores of the three organs was taken as the total score. Then, one half of the spleen or lung was cut and placed in a grinding tube, 3 mL of normal saline was added, and the tissue was ground evenly. Then, 1 mL of lung homogenate was added to 1.0 mL of 4% sulfuric acid and the mixture was incubated at room temperature for 15 min to eliminate any other miscellaneous bacteria. The acid in the mixture was then neutralized with 2 mol/L of sodium hydroxide solution. The spleen homogenate was not processed. Next, 10-fold serial dilutions were conducted according to a ratio of 0.5 mL of spleen/lung homogenate to 4.5 mL of normal saline, and different dilutions were inoculated according to the degree of organ lesions. Each dilution was inoculated into two tubes of L–J medium and cultured at 37 °C for 4 weeks before colony counting.

### 2.9. Histopathological Examination

The livers, lungs, and spleens of the guinea pigs in all the groups were fixed in 10% paraformaldehyde solution, and then embedded in paraffin after dehydration. The tissue was sectioned at an approximate thickness of 4 μm, and lesions were observed under a microscope after hematoxylin and eosin (HE) staining.

### 2.10. Drug Sensitivity Testing of Phenotypes

According to the WHO Guidelines for the Surveillance of Tuberculosis Drug Resistance (sixth edition) [21,22], the drug resistance of the spleen bacterial cultures of the INH-RFP (2 w) (group 5), INH-RFP (4 w) (group 6), and NS (group 9) was detected. The positive control bacteria were resistant to both INH and RFP. The final concentrations of INH and RFP in the L–J medium were 0.2 μg/mL and 40 μg/mL, respectively. The determination criterion for drug resistance was as follows: (the number of colonies in drug-containing medium/the number of colonies in control medium) × 100% > 1%.

### 2.11. Drug Sensitivity Testing of Genotypes

According to the instructions of the *M.tb* rifampicin resistance mutation detection kit (fluorescent PCR melting curve method) and the *M.tb* isoniazid resistance mutation detection kit (fluorescent PCR melting curve method), the drug resistance of the spleen bacterial culture in the INH-RFP (2 w) (group 5), INH-RFP (4 w) (group 6), and NS (group 9) groups was detected.

### 2.12. Statistical Analysis

Statistical analyses were conducted using GraphPad Prism 9.0 (GraphPad Software, San Diego, CA, USA), the Shapiro–Wilk test was used for the normal distribution test, and the statistical significance of the difference between the two groups was assessed using a *t*-test, with a one-way ANOVA or two-way ANOVA for the analysis of the differences between multiple groups. The results are expressed as the mean ± SEM. Differences were considered significant at */^#^ *p* < 0.05, **/^##^ *p* < 0.01, ***/^###^ *p* < 0.001.

## 3. Results

### 3.1. Effects of Immunotherapy on the General Condition of Guinea Pigs

**Figure 2:**
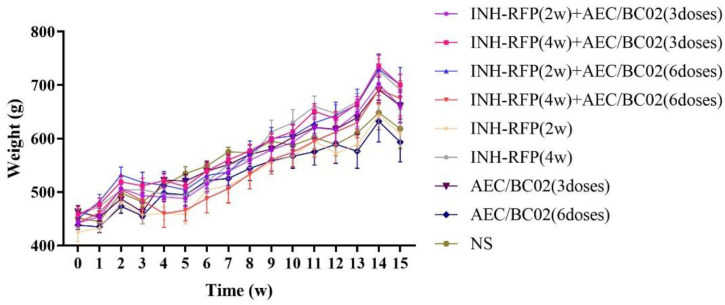
Changes in guinea pig body weight over time (mean ± SEM).

Following the *M.tb* infection of the guinea pigs, some animals showed pathological nodular swelling at the injection site. During the whole experimental period, there was no obvious abnormality in the guinea pigs in terms of their fur or general condition, or their activity levels, and their body weight gradually increased with their feeding time, as shown in Figure 2. During week 2, the body weight of the guinea pigs increased slowly, potentially as a side effect of the antibiotics and an animal stress response; however, their body weight returned to normal after the end of the intragastric administration. During week 15, one week before dissection, the body weight of the guinea pigs decreased slightly as a result of fasting. Overall, the binary analysis of variance showed that time had a significant effect (*F* = 353.1, *p* < 0.0001), while the therapeutic regimen had no significant effect; that is, there was no significant difference in the weight of the guinea pigs between the different groups (*F* = 1.585, *p* = 0.1564).

### 3.2. Effects of Different Immunotherapy Procedures on the Gross Pathological Score and Bacterial Loads

#### 3.2.1. Effect of Different Administration Cycles

**Figure 3:**
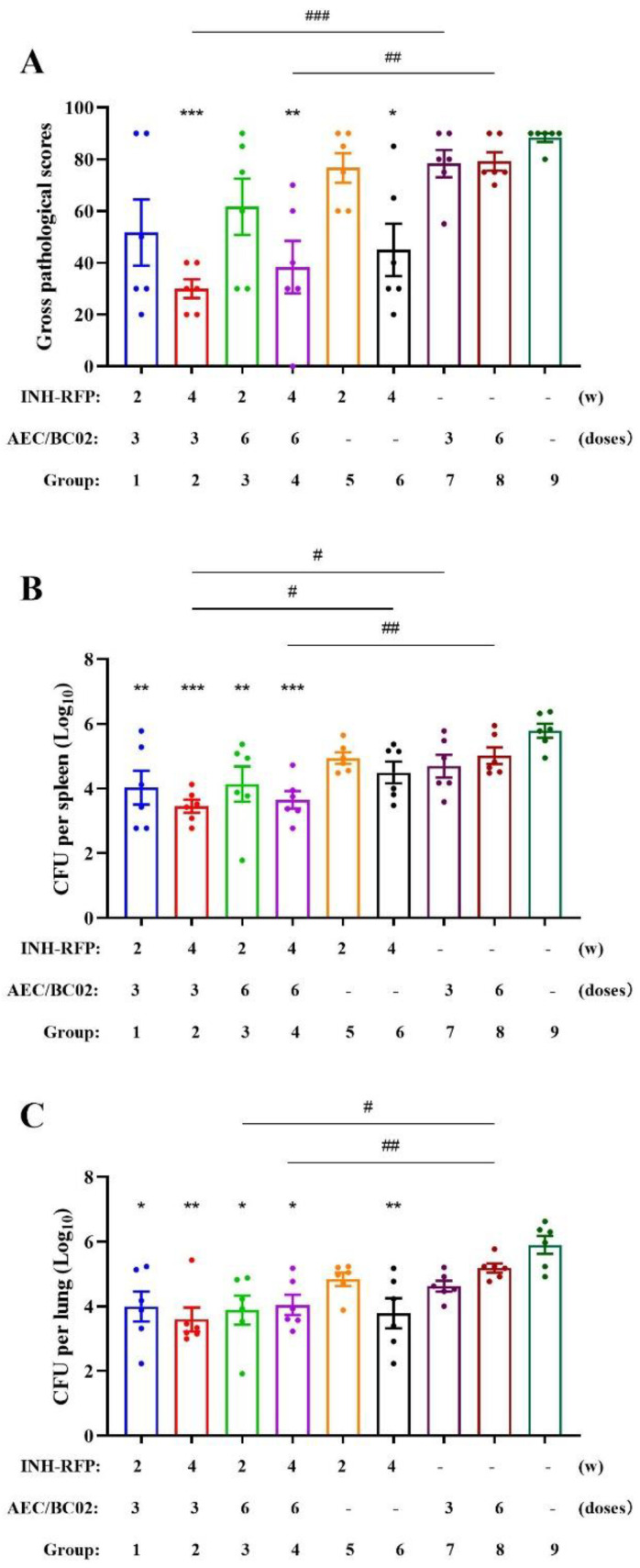
Effects of different immunotherapy procedures on the gross pathological score and bacterial loads (mean ± SEM). (**A**) shows the gross pathological scores for the spleen, lung and liver; (**B**) shows the bacterial loads of the spleen tissue; (**C**) shows the bacterial loads of the lung tissue. * indicates that the experimental group was compared with the NS group, and ^#^ indicates that the experimental group was compared with the designated group. */^#^ *p* < 0.05, **/^##^ *p* < 0.01, ***/^###^ *p* < 0.001.

The protective effect of the AEC/BC02 vaccine 3 against guinea pigs was analyzed by comparing groups 1, 2, 5, 6, 7, and 9 after the administration of isoniazid–rifampin tablets for different periods of time (2 or 4 weeks). The gross pathological scores, as shown in Figure 3A, were significantly lower in INH–RFP (4 w) + AEC/BC02 (three doses) (group 2) and INH–RFP (4 w) (group 6), compared with the NS (group 9). In addition, the gross pathological score of group 2 was significantly lower than that of AEC/BC02 (three doses) (group 7) and lower than that of INH–RFP (2 w) + AEC/BC02 (three doses) (group 1, 22 ± 10 scores reduction). As shown in Figure 3B,C, the number of viable bacteria in the spleen and lung of each treatment group was lower than that of the negative control (group 9), with significant differences being detected between groups 1, 2, and 9. In the spleen, the number of viable bacteria in the group 2 was significantly lower than that of group 7. In the lung, the number of viable bacteria in the 4-week group (group 6) was also significantly lower than that in group 9, while the viable bacteria in the 2-week group (group 5) were lower than in the NS group, but there was no statistically significant difference.

The protective effect of the AEC/BC02 vaccine 6 against the guinea pigs was analyzed by comparing groups 3, 4, 5, 6, 8, and 9 after the administration of isoniazid–rifampin tablets for different periods of time (2 or 4 weeks). Similar to the results with three doses of vaccine, the gross pathological score of INH-RFP (4 w) + AEC/BC02 (six doses) (group 4) were significantly lower than that of the group 9. The gross pathological score of group 4 was significantly lower than that of AEC/BC02 (six doses) (group 8) and lower than that of INH-RFP (2 w) + AEC/BC02 (six doses) (group 3, 23 ± 12 scores reduction). The number of viable bacteria in the spleen and lung of group 3 and group 4 were significantly lower than that of the group 9. The amount of spleen bacteria in group 4, and the amount of spleen and lung bacteria in groups 3 and 4, were significantly lower than those in the single 6-needle immunization group.

In conclusion, the results of this comparison showed that the administration of isoniazid–rifampin tablets followed by a vaccine can significantly improve the protective effect of a high dose *M.tb* infection in guinea pigs compared with the group immunized with a vaccine alone. The effect of isoniazid–rifampicin tablets was poor after only 2 weeks of administration, but after 4 weeks of administration, isoniazid–rifampicin tablets could effectively reduce the gross pathological score and the number of bacteria in the organs. The protective effect in the 4-week group was higher than that in the 2-week group.

#### 3.2.2. Effect of Different Immunization Times

The protective effect of different doses (three or six doses) of the AEC/BC02 vaccine on the guinea pigs was analyzed by comparing groups 1, 3, 5, 7, 8, and 9 after 2 weeks of administration of isoniazid–rifampin tablets. As shown in Figure 3A, the gross pathological scores of INH-RFP (2 w) + AEC/BC02 (three doses) (group 1) and INH-RFP (2 w) + AEC/BC02 (six doses) (group 3) were lower than that of the NS control group (group 9). The lesion index of group 1 was lower than that of group 3 (10 ± 17 scores reduction). In addition, the gross pathological score of groups 1 and 3 was lower than that of group 5 (25 ± 10 scores reduction, and 15 ± 15 scores reduction). As shown in Figure 3B,C, the number of viable bacteria in the spleen and lung of group 1 and group 3 was significantly lower than that of group 9, and the results of group 1 and group 3 were similar.

The protective effects of different doses (three or six doses) of the AEC/BC02 vaccine in guinea pigs were analyzed by comparing groups 2, 4, 6, 7, 8, and 9 after 4 weeks of administration of isoniazid–rifampin tablets. INH-RFP (4 w) + AEC/BC02 (three doses) (group 2), INH-RFP (4 w) + AEC/BC02 (six doses) (group 4), and INH-RFP (4 w) (group 6) showed significantly lower scores than those in group 9, and the decrease in group 2 was more significant than that in groups 4 and 6. Compared with group 9, groups 2 and 4 showed significantly decreased numbers of viable bacteria, and the number of viable bacteria in the organs was slightly decreased in the 3-needle group compared with the 6-needle group. In addition, the number of viable bacteria in the spleens of group 6 was significantly higher than that of group 2, and higher than that of group 4 (0.85 ± 0.45 CFU increase).

In conclusion, in the guinea pigs infected with high dose *M.tb*, the immune vaccine further reduced the organ lesion score and the number of bacteria, compared with the single administration group, indicating that the vaccine had a certain protective effect in guinea pigs. The protective effect was slightly improved in the three-dose group compared with the six-dose group.

### 3.3. Effects of Different Immunotherapy Procedures on Histological Changes

The examination of the pathological sections of the liver, spleen, and lung revealed typical tuberculous granulomatous lesions in all the groups of guinea pigs (Table 3). Compared with the NS control group, the severity of the lesions in the different drug groups was reduced. In both INH-RFP (4 w) + AEC/BC02 (three doses) (group 2) and INH-RFP (4 w) + AEC/BC02 (six doses) (group 4), the degree of organ lesions was similar and was slightly lower than in the vaccine groups (AEC/BC02 (three doses) (group 7), AEC/BC02 (six doses) (group 8)) or the chemotherapy groups (INH-RFP (2 w) (group 5), and INH-RFP group (4 w) (group 6)).

### 3.4. Effect of Short-Term Chemotherapy on Bacterial Resistance

The results obtained using the drug sensitivity test proportion method showed that the splenic bacterial cultures of the INH-RFP (2 w) group (group 5), INH-RFP (4 w) group (group 6), and NS group (group 9) were sensitive to isoniazid and rifampicin, and there was no resistance to these agents.

The results obtained using the *M.tb* rifampicin resistance mutation detection kit (fluorescent PCR melting curve method) and the *M.tb* isoniazid resistance mutation detection kit (fluorescent PCR melting curve method) also showed that the spleen cultures from the NS (group 9), INH-RFP (2 w) (group 5), and INH-RFP (4 w) (group 6) groups were all rifampicin and isoniazid sensitive.

These results indicated that isoniazid- or rifampin-resistant strains did not occur in guinea pigs treated with isoniazid–rifampin tablets at the doses used in this study over 2 or 4 weeks.

## 4. Discussion

The ability of *M.tb* to lie dormant and persist in humans without producing the disease limits the eradication of tuberculosis. LTBIs are widespread, and it is estimated that 5% to 10% progress to active tuberculosis [23]. Therefore, the inhibition and treatment of an LTBI is currently the focus of tuberculosis research. Chemotherapy is an important strategy for the control of tuberculosis. Because of its compliance and toxicity problems, as well as the high risk of reinfection in high-incidence countries after the end of chemotherapy, and the existence of isoniazid-resistant strains, chemotherapy is highly ineffective. Vaccines, as a type of immunotherapy, can effectively shorten the treatment time and potentially reduce the recurrence of the disease.

At present, the RUTI vaccine [24] is being studied worldwide and has achieved considerable results in animal models and clinical trials [25,26,27,28,29]. The basic principle of RUTI immunotherapy is to first use the bactericidal properties of chemotherapeutics to eliminate actively-propagating *M.tb* and prevent the reactivation of latent bacilli that are trying to reproduce. In addition, chemotherapeutics can eliminate the outermost layer of foamy macrophages outside granulomas and reduce the local inflammatory response, thereby reducing the possibility of the Koch phenomenon caused by *M.tb* antigens during treatment [30,31]. After chemotherapy, the inoculation of RUTI may reduce the probability of the regrowth of the remaining latent bacilli. The vaccine analyzed in this study is a novel subunit vaccine, AEC/BC02, with a similar therapeutic principle to RUTI [24]. Our previous studies have shown that an AEC/BC02 immunization after short-term chemotherapy can induce a certain degree of humoral and cellular immunity in the mouse latent infection model, mainly Th1 immunity, and induced a protective effect in both mice and guinea pigs [19,32]. At present, the AEC/BC02 vaccine has completed phase I clinical trials, and after six doses of intramuscular immunization in healthy volunteers, the results showed an acceptable safety profile and a certain degree of immunogenicity (data unpublished). The aim of our study was to optimize the best treatment for phase II clinical trials. We observed the general condition, gross pathological score, viable count, and pathological results of animals by comparing different chemotherapy cycles and different immune injections to determine the optimal treatment plan. The results showed that the guinea pigs subjected to our treatment protocol remained healthy, with no mortality, and showed a normal increase in the body weight, indicating that the combination of short-term chemotherapy and the AEC/BC02 vaccine immunization offered a good safety profile.

The gross pathological scores and viable counts following a short-term treatment with isoniazid–rifampin tablets alone were lower than those of the control group, and the 4-week chemotherapy treatment resulted in significantly lower gross pathological scores and viable counts than the 2-week treatment. In the AEC/BC02 vaccine alone group, the organ lesion score and viable count also decreased slightly, after either three or six injections, but there was no difference compared with the saline control group, and the effect was less pronounced than with chemotherapy alone. This indicated that the vaccine alone did not produce a protective effect in guinea pigs, which is consistent with the findings with the RUTI vaccine [24]. After treatment with isoniazid–rifampin tablets combined with AEC/BC02, the indexes were lower than those of the chemotherapy alone group and the immunization alone group. In the combined immunotherapy regimen, 4 weeks of treatment with chemotherapy and three or six doses of an AEC/BC02 immunization could achieve a better immune protection, with a significant decrease in the organ lesion score and viable count, and three doses of vaccine immunization was slightly preferable to six doses. The same trend was found for the organ pathology results with each treatment group.

In 2021, 2.4 million of bacteriologically confirmed tuberculosis patients worldwide were tested for rifampicin resistance, and a total of 166,991 cases of drug-resistant tuberculosis were detected [1]. The emergence of drug-resistant tuberculosis results from irrational drug use, poor patient compliance, poor drug supply or quality, and differences in the metabolism and nutrition [33]. Currently, the rise in drug-resistant tuberculosis presents a challenge in terms of treatment and threatens the control of tuberculosis worldwide [34]. Therefore, when studying new immunotherapeutic strategies, the use of chemotherapy to reduce the emergence of drug resistance should be considered. In this study, phenotypic and genotypic drug sensitivity tests were carried out in the chemotherapy alone group, and the results showed that there was no drug resistance, indicating that the risk of drug resistance was low after 2 or 4 weeks of the administration of isoniazid–rifampin tablets.

In conclusion, after guinea pigs were infected with high dose virulent strains of *M.tb*, the intragastric administration of isoniazid–rifampin tablets for 4 weeks to inhibit Koch’s reaction, combined with three or six doses of the freeze-dried recombinant tuberculosis vaccine (AEC/BC02), obtained a good immune protective effect compared with other administration methods. This was evidenced by significantly reduced pathological changes in animal organs and reduced viable bacterial loads in the spleen and lung. Moreover, three injections of vaccine immunization obtained slightly improved results compared with six injections, thereby potentially shortening the necessary course of immunization and improving the compliance to the treatment. At the same time, the drug resistance of *M.tb* was not detected after 2 or 4 weeks of the administration of dual antibiotics, indicating that the risk of drug-resistant *M.tb* was low after a short-term treatment with multiple antibiotics, further demonstrating the feasibility of short-term chemotherapy followed by the AEC/BC02 vaccine immunization regimen.

## Figures and Tables

**Table 1:** Description of the experimental guinea pig groups.

Group Number	Group	n
1	INH-RFP (2 w) + AEC/BC02 (3 doses)	6
2	INH-RFP (4 w) + AEC/BC02 (3 doses)	6
3	INH-RFP (2 w) + AEC/BC02 (6 doses)	6
4	INH-RFP (4 w) + AEC/BC02 (6 doses)	6
5	INH-RFP (2 w)	6
6	INH-RFP (4 w)	6
7	AEC/BC02 (3 doses)	6
8	AEC/BC02 (6 doses)	6
9	NS	6

**Table 2:** Criterion for the gross pathological score of the liver, spleen and lung.

Degree of Lesion	Liver	Spleen	Lung
None	0	0	0
Mild	10	10	10
Moderate	20	20	20
Severe	25	35	30

**Table 3:** Histopathologic examination of typical spleen, lung and liver tissues of guinea pigs in each group (HE staining, 100× magnification).

Group	Spleen	Lung	Liver
INH-RFP (2 w) + AEC/BC02 (3 doses)	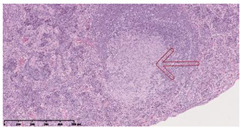 Arrows indicate granulomatous lesions	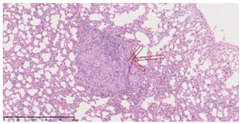 Arrows indicate granulomatous lesions	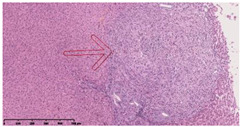 Arrows indicate granulomatous lesions
INH-RFP (4 w) + AEC/BC02 (3 doses)	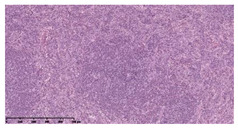 No granulomatous lesions	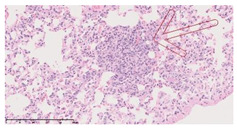 Arrows indicate granulomatous lesions	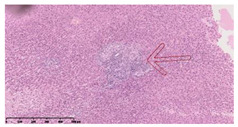 Arrows indicate granulomatous lesions
INH-RFP (2 w) + AEC/BC02 (6 doses)	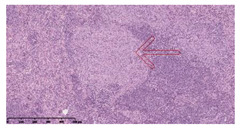 Arrows indicate granulomatous lesions	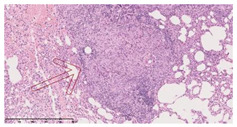 Arrows indicate granulomatous lesions	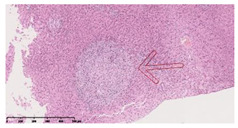 Arrows indicate granulomatous lesions
INH-RFP (4 w) + AEC/BC02 (6 doses)	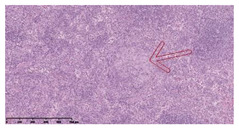 Arrows indicate granulomatous lesions	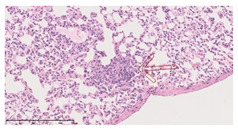 Arrows indicate granulomatous lesions	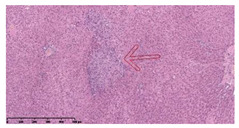 Arrows indicate granulomatous lesions
INH-RFP (2 w)	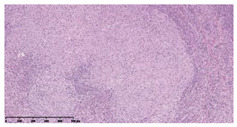 Extensive granulomatous lesions	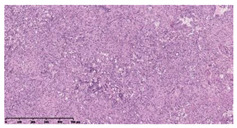 Extensive granulomatous lesions	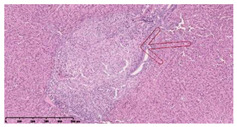 Arrows indicate granulomatous lesions
INH-RFP (4 w)	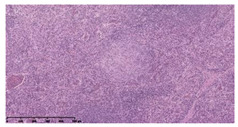 Granulomatous lesions in the middle	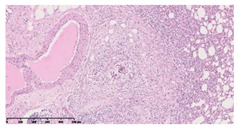 Extensive granulomatous lesions	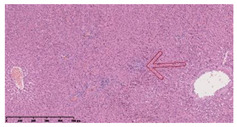 Arrows indicate granulomatous lesions
AEC/BC02 (3 doses)	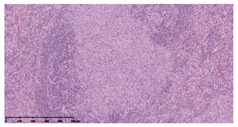 Extensive granulomatous lesions	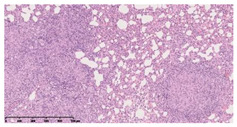 Large granulomas lesions on both sides	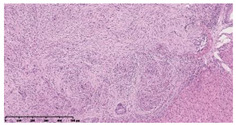 Extensive granulomatous lesions
AEC/BC02 (6 doses)	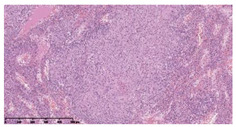 Extensive granulomatous lesions	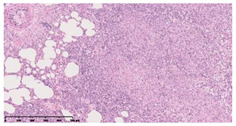 Extensive granulomatous lesions	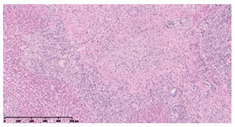 Extensive granulomatous lesions
NS	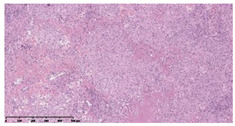 Extensive granulomatous lesions	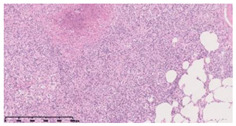 Extensive granulomatous lesions	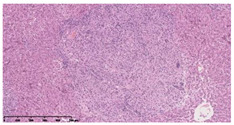 Extensive granulomatous lesions

## Data Availability

The datasets generated for this study are available on request to the corresponding author.
